# Musical feedback system Jymmin^®^ leads to enhanced physical endurance in the elderly—A feasibility study

**DOI:** 10.3389/fspor.2022.915926

**Published:** 2022-08-11

**Authors:** Kathrin Rehfeld, Thomas Hans Fritz, Alexander Prinz, Lydia Schneider, Arno Villringer, Kerstin Witte

**Affiliations:** ^1^Institute for Sport Science, Otto-von-Guericke University Magdeburg, Magdeburg, Germany; ^2^Department of Neurology, Max Planck Institute for Human Cognitive and Brain Sciences, Leipzig, Germany; ^3^Institute for Psychoacoustics and Electronic Music (IPEM), Ghent, Belgium

**Keywords:** Jymmin®, seniors, physical exercise, aging, musical feedback system

## Abstract

**Background and objectives:**

Active music-making in combination with physical exercise has evoked several positive effects in users of different age groups. These include enhanced mood, muscular effectivity, pain threshold, and decreased perceived exertion. The present study tested the applicability of this musical feedback system, called *Jymmin*^®^, in combination with strength-endurance exercises in a population of healthy older adults.

**Research design and methods:**

Sixteen healthy, physically inactive older adults (5 males, 11 females) at the mean age of 70 years performed physical exercise in two conditions: A conventional work-out while listening passively music and a *Jymmin*^®^ work-out, where musical sounds were created with one's work-out movements. According to the hypothesis that strength-endurance is increased during musical feedback exercise, parameters relating to strength-endurance were assessed, including *exercise duration, number of repetitions, perceived exertion* (RPE), and *participants' mental state* (Multidimensional Mood State Questionnaire; MDMQ).

**Results:**

Results show that participants exercised significantly longer while doing *Jymmin*^®^ (*Mdn* = 248.75 s) as compared to the *conventional work-out* (*Mdn* = 182.73 s), (Z = 3.408, *p* = 0.001). The RPE did not differ between *conventional work-out* and the *Jymmin*^®^ condition, even though participants worked out significantly longer during the *Jymmin*^®^ condition (Mdn = 14.50; Z = −0.905; *p* = 0.366). The results of the MDMQ showed no significant differences between both conditions (Z = −1.037; *p* = 0.300).

**Discussion and implications:**

Results show that participants could work out longer while showing the same perceived exertion, relating to increased physical endurance. Music feedback work-out encouraged a greater degree of isometric contractions (muscle actively held at fixed length) and, therefore, less repetitions in this condition. In addition to the previously described effect on muscle effectivity, this non-stereotypic contraction pattern during music feedback training may have enhanced endurance in participants supporting them to better proportion energetic reserves during training (pacing).

**Clinical trial registration:**

Identifier: DRKS00023645.

## Introduction

The benefits of physical activity for health in older adults are well established (Netz et al., 2005; Windle et al., 2010; Taylor, 2014). Hence, physical exercise is a crucial factor in promoting the development and maintenance of a functional ability that enables wellbeing in older age. Music has become a second important key factor contributing positively to normal aging, rehabilitation and therapy (Schneider et al., 2007; Forsblom et al., 2009). Listening to music with no additional physical work-out can promote physiological effects (Shimizu et al., 2013). Meditative music for instance decreases activation of the sympathetic nervous system and may help to reduce anxiety and prevents stress-induced systolic blood pressure and heart rate (Knight and Rickard, 2001; Marks and Landaira, 2015). Especially several components of music, like tempo, melody and rhythm have an impact on the central nervous system (Karageorghis et al., 1999; Simpson and Karageorghis, 2006). Rasteiro et al. (2020) investigated the effect of preferred music on anaerobic threshold determination in an incremental running test, physiological responses and perceived exertion in men and women. Since there was no significant effect of music on any of the variables, preferred music is not prerequisite.

Linking music to physical exercise can reduce perceived exertion, improve mood, decrease anxiety and depression, and moderate one's feelings during high-intensity exercise positively (Karageorghis and Priest, 2012). For example, Hayakawa et al. (2000) showed reduced feelings of fatigue when physically exercising with music compared to a non-music work-out. Listening to music during exercise can also help to minimize distractions due to environmental noise and improve task-associated concentration (Ruscello et al., 2014). The meta-analysis of Terry et al. (2020) quantified the effects of music listening in exercise and sport and showed beneficial effects on affective valence, physical performance, perceived exertion and oxygen consumption.

Numbers of studies found that especially synchronous music improves performance in motor tasks since synchronous music influences the economy by lower oxygen consumption and blood lactate levels (Simpson and Karageorghis, 2006; Terry et al., 2012). Music-based exercise programs have accordingly been shown to lead to improved gait, balance, and a lower risk of falls among a group of community-dwelling elders (Brown and de Bruin, 2011) and further improved age-correlated motor abilities like balance and reaction times (Rehfeld et al., 2019). Therefore, synchronizing physical exercise to music seems to be a powerful compound in healthy aging. Music is mainly employed passively to diminish perceived exertion (Potteiger et al., 2000; Yamashita et al., 2006) and support movement in rehabilitation (Brown and de Bruin, 2011; Schaefer, 2014). It is commonly used as an auditory cue to foster movement coordination. However, music can also be integrated as an active music making part in therapy and prevention programs. Usually, active music making involves participants playing instruments and using their voices. The use of instruments is structured to involve all the sensory organs and the rhythmic and melodic components of music to obtain specific motor and emotional responses. Thus, combining movement and stimulation of different sensory pathways (auditory, tactile, multiple sensory stimulations) established emotional quality (Pacchetti et al., 2000), commitment to exercising, and even having a higher impact than passive music. To combine a high quality of physical exercise and active music making, musical feedback systems have been developed to achieve the beneficial effects of physical exercise and music.

Sports movements can be mapped to the phases of the beat of the music for instance. Because music usually has a relatively steady beat, this type of mapping only works for cyclic movements that are stereotypical and repetitive, such as during ergometer training or running. In such a case, the tempo and sometimes the degree of instrumentation of the music feedback are related to the phase of the cyclic movement (van der Vlist et al., 2011; Moens et al., 2014). An endurance exercise experiment used a musical feedback system called moBeat to map cyclic movement to music with an ergometer (van der Vlist et al., 2011). The authors showed a positive effect on intrinsic motivation and attentional focus but not on perceived exertion (van der Vlist et al., 2011).

Another musical feedback system called Jymmin^®^ can be applied for resistance training and was developed by Fritz et al. (2013a). Fritz et al. (2013b) demonstrated that young adults (28 years old) showed significantly better results in oxygen consumption, perceived exertion and force when exercising with musical feedback in a resistance training compared to regular work-out (listening passively to music). The work-out under the condition musical feedback had also enhancing effect on mood compared to work-out with passive music listening in young adults (Fritz et al., 2013a). The musical feedback system Jymmin^®^ seems to be a promising new approach to combine the benefits of active music making and physical training. Especially for elderly adults, it could be a beneficial method to counteract age-correlated processes, because they are exposed to a complex sensory and motor surrounding, which stimulate somatosensory and auditory sensations. The combination of coordinating the movement execution and the musical sound with more than just one participant in one training session increases the level of attention to several stimuli and could be comparable to the effects of dancing (Müller et al., 2017; Rehfeld et al., 2018) only in a resistance training context. To the best of our knowledge this approach has not been tested for older adults. Hence, this feasibility study aimed to test a musical feedback system with healthy elderly adults in strength-endurance exercises. We hypothesize that the training with a music feedback system would enhance physical training parameters like exercise duration and number of repetitions, compared to conventional work-out (passive music). Furthermore, we assume that a musical feedback system will distract participants more from subjective perceived exertion compared to conventional work-out (passive music).

## Materials and methods

### Subjects

Sixteen healthy elderly adults (11 females; 5 males) with a mean age of 70.6 years (SD ± 3.9 years) participated in this study. The participants were recruited by advertisement in local newspapers. None of the participants were regularly physically active. The grip force of all participants was assessed, which relates to physical fitness in the elderly. The elderly females revealed a grip strength mean-value of 290 N and males achieved 412 N. These mean-values represent normative performances of grip force in this age group and sexes (Steiber, 2016). Exclusion criteria were severe cardiovascular diseases such as cardiac arrhythmia, pulmonary disease, or untreated, higher grade hypertension. To determine medical status as well as former severe diseases participants had to fill in a health survey. However, our participants did not receive financial reimbursement and informed consent was obtained. The research protocol conformed to principles of the Declaration of Helsinki and was approved by the Ethic Committee of Otto von Guericke University Magdeburg (Germany: registration number: 193/18).

### Musical feedback system Jymmin^®^

Jymmin^®^ is developed by Fritz et al. (2013a) and is a mixture between jamming and gym. In Jymmin^®^, a sensor can be equipped to several fitness devices to modulate musical audio feedback (Fritz et al., 2013a). The movement of the sensor equipped fitness device is mapped to a musical composition software (Ableton Live 8). The deflection of the fitness device corresponds to musical parameters of an acoustic feedback signal. In the musical composition software, Fritz and colleagues prepared a series of musical loops, which are set to repeat and temporally synchronize at a constant tempo of 120 beats per minute (bpm). Already relatively small movements in the centimeter range create a perceivable musical effect. Interestingly, several fitness devices equipped with sensors can be interactively combined into a holistic musical piece.

### Study design

This feasibility study aimed to test a musical feedback system called Jymmin^®^, which was used in combination with a strength-endurance fitness machine called the Body-Spider (Koopera Inc.). The Body-Spider is a mobile training device with pull robes. The exercises can be performed both sitting and standing, and all muscle groups can be trained.

**Figure 1:**
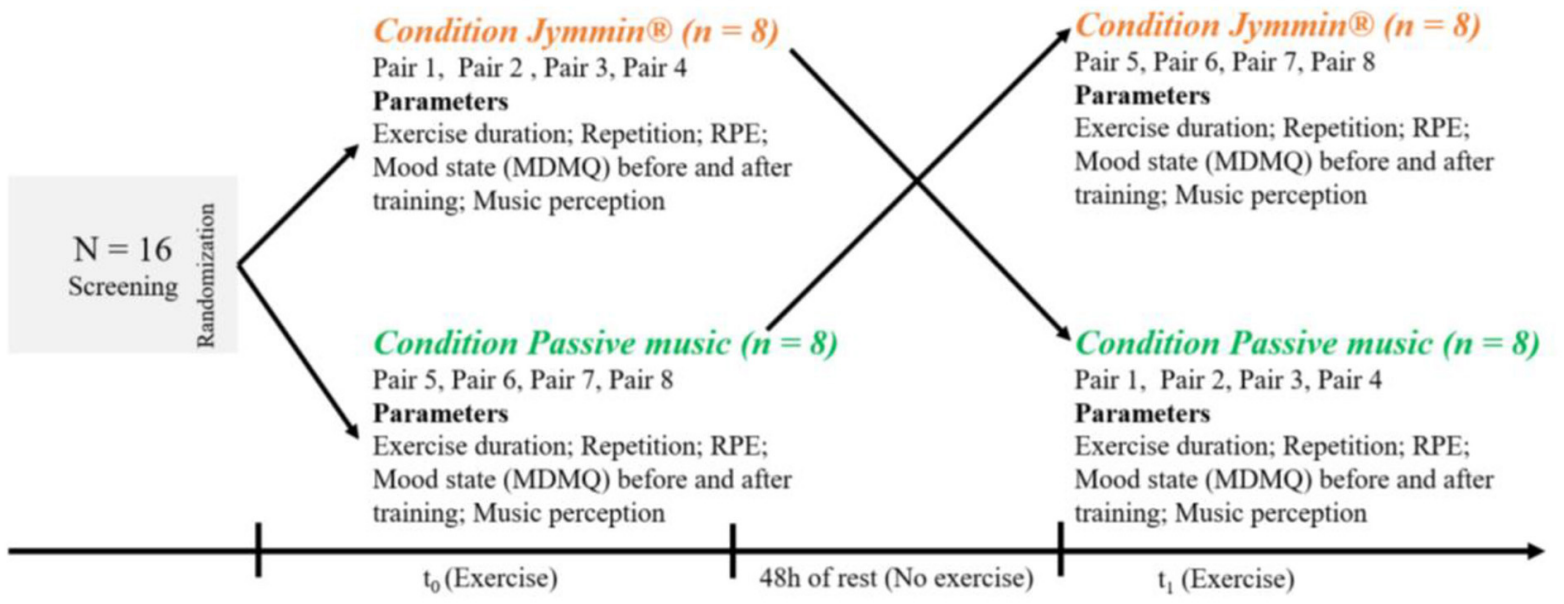
Within-subject crossover design to test musical feedback system Jymmin^®^. Annotation: RPE = Rate of Perceived Exertion; Two elderly adults work-out in pairs to the same time.

In a within-subjects crossover design (Figure 1), participants executed strength-endurance exercises on 2 days within 1 week. The exercises were performed during two different experimental conditions, condition 1 (conventional work-out) and condition 2 (Jymmin^®^-music feedback). In the conventional work-out (cond 1) participants worked out while listening to music but could not directly influence the music composition through their movement. In condition 2 (Jymmin^®^), participants influenced and controlled the musical pieces of the Jymmin^®^ software through their movements. Each exercise was linked to a different piece of music. In both conditions, the same pieces of music were used for a given exercise to precisely control the influence of the music. The sequence in how participants performed with or without music control was balanced. Eight participants performed the first training with condition one, followed by condition two, and eight participants performed it vice versa. Participants had 72 h of rest between both training days.

### Procedure

**Figure 2:**
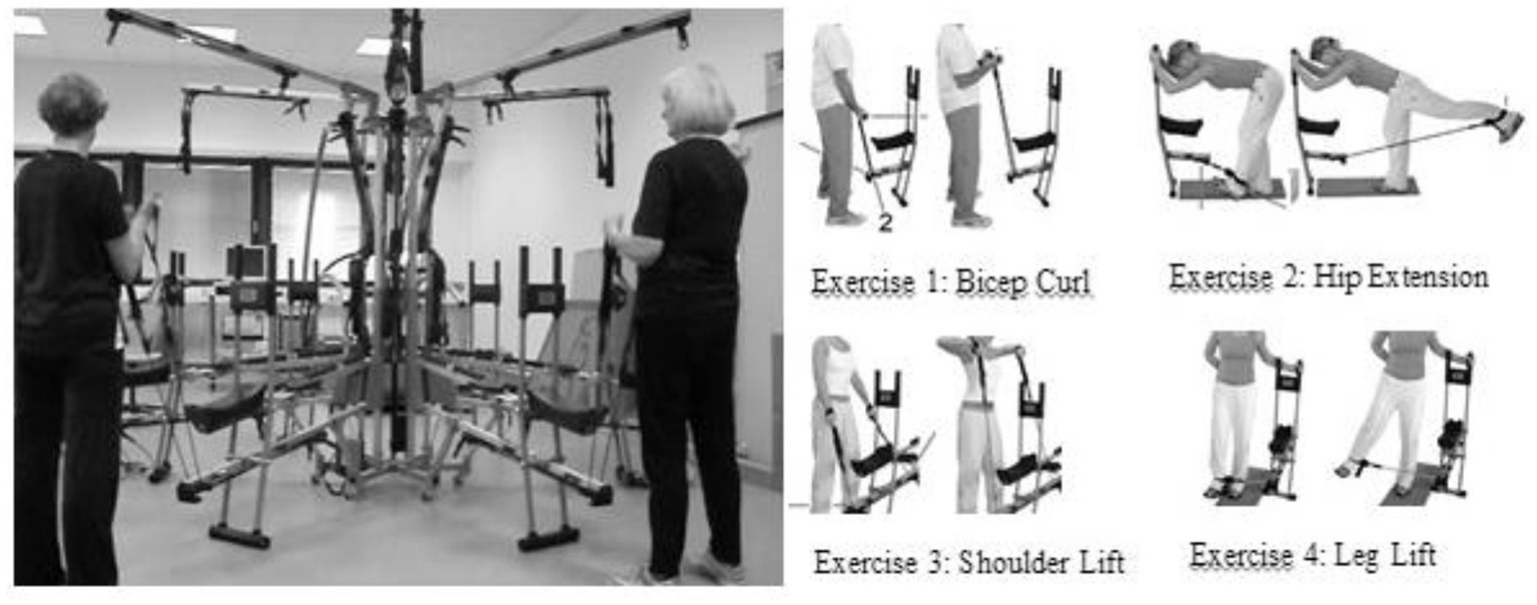
Body-Spider^®^ equipped with sensors and the four exercises that were conducted.

For the strength endurance program, four predefined exercises from the manufacturer were used: Bicep Curl, Hip Extension, Shoulder Lift and Leg Lift (Figure 2). The musical feedback was designed such that two fitness exercises performed by two participants created musical sounds that may be combined to create a holistic musical piece at a constant tempo of 120 beats per minute (bpm). The movement of the participants at the sports equipment was recognized by a sensor attached to the exercise machine and converted into music, creating acoustic feedback using specially developed sensor hardware and software (Jymmin^®^). The tempo of 120 bpm was chosen according to previous studies suggesting human movement and rhythmical perception are both bound to the same optimal frequency of 120 bpm (Kay et al., 1987; Schneider et al., 2010).

After a 10-min warm-up, the participants were told to perform each of the four exercises until they could no longer manage a repetition. Between the exercises, a break with a length of 450 s was completed. The participants always performed the exercises in pairs. Instructors were always present to supervise the correct movement execution with the fitness machine.

### Outcome parameters

Various physical and psychological parameters were tested: Duration of exercise, number of repetitions, subjectively perceived effort, the mental state before and after each training period, and subjective response to music.

#### Duration of exercise

The participants were instructed to exercise as long as possible. If the subject discontinued the exercise, the time was noted. The duration of each exercise was measured with a stopwatch.

#### Number of repetitions

The number of repetitions was recorded by video cameras. To estimate the number of repetitions, the number of complete repetitions in 1 min was extrapolated to the total duration of the exercise.

#### Rating of perceived exertion

The perceived exertion of the subjects was assessed using the Borg Scale. The scale ranges from a minimum value of 6 (no exertion) to a maximum value of 20 (maximum exertion). The values were documented every 90 s during the exercise performance and immediately after the abortion of the exercise. Thus, for each exercise of each condition, a mean value of effort was calculated and then analyzed.

#### Multidimensional mood state questionnaire

The MDMQ by Steyer et al. (2003) is a mental state assessment instrument that measures the current mental state with three bipolar designed subscales (good/bad mood, alertness/fatigue, calmness/restlessness) with a total of 12 items. Each subscale consists of four adjectives, of which two belong to the negative (e.g., bad uncomfortable) and two to the positive (e.g., well satisfied) pole. These items are rated on a five-point Likert-scale with one not at all and five very. The MDMQ is designed for adolescents as well as adults and can be used to evaluate the progress of mood influencing therapies and interventions.

#### Subjective music perception

After each condition, participants in the conventional work-out (cond 1) and Jymmin^®^ (cond 2) were asked to complete a Likert scale in which they expressed their personal opinions about the music. The scale ranged from 0 (not my taste) to 10 (exactly my taste).

### Statistical analysis

#### Physical and psychological data

The obtained data were analyzed with non-parametric tests using SPSS 22 (IBM) for all 16 participants on physical (duration and endurance) as well as psychological (exertion, mood, and pleasantness of music) parameters. A Wilcoxon Signed Ranks Test for dependent samples was applied to test whether the physical or psychological parameters differed between the conventional work-out condition and the Jymmin^®^ condition. Sequence effects, using the Mann-Whitney U Test were determined to evaluate if the order of conditions affected the results. Effect sizes were calculated: *r* < 0.4 = strong effect; *r* < 0.25 = moderate effect; *r* < 0.1 = low effect.

## Results

### Physical and psychological parameters

**Figure 3:**
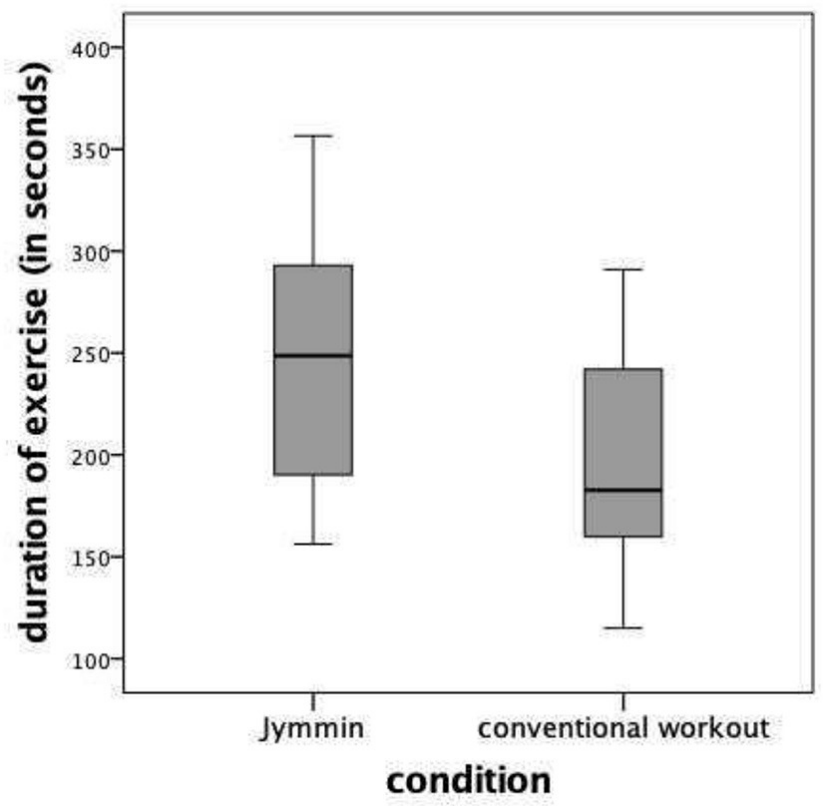
Duration of the exercises during Jymmin and conventinal workout.

The results showed that participants exercised significantly longer during Jymmin^®^ (Mdn = 248.75 s) as compared to the conventional workout (Mdn = 182.73 s), Z = 3.408, *p* = 0.001 (see Figure 3). The results revealed a strong effect size of *r* = 0.85. No sequence effect was found for duration of exercise [for grouping variable: sequence: U = −1.323; *p* = 0.210 (passive music); U = −1.217; *p* = 0.252 (Jymmin)].

The number of repetitions divided by the duration of exercise revealed a significant difference between the Jymmin^®^ condition (Mdn = 0.31) as compared to the conventional work-out condition (Mdn = 0.43). The number of repetitions divided by the duration of exercise was significantly higher during conventional work-out (Z = −3.361, *p* = 0.001). The effect size is *r* = 0.9. No sequence effect was found for the number of repetitions [for grouping variable: sequence: U = −1.641; *p* = 0.114 (passive music); U = −0.582; *p* = 0.606 (Jymmin)].

The results of perceived exertion indicated no significant differences between the Jymmin^®^ condition (Mdn = 14.00) as compared to the conventional work-out condition (Mdn = 14.50; Z = −0.905; *p* = 0.366). The effect size is low (*r* = 0.23). No sequence effect was found for perceived exertion scores [for grouping variable: sequence: U = – 0.265; *p* = 0.8.73 (passive music); U = −0.530; *p* = 0.606 (Jymmin)].

The results of the MDMQ showed no significant difference between the difference of pre-intervention mood scores and post-intervention mood scores for the conventional work-out condition (Mdn = 0.50) and the Jymmin^®^ condition (Mdn = 0.50) applying a Wilcoxon Signed Rank Test (Z = −1.037; *p* = 0.300). A medium effect size was calculated (*r* = 0.26).

The perception of the musical piece during the conventional work-out (Mdn = 5.00) as compared to the Jymmin^®^ condition (Mdn = 6.00) did not differ significantly (Z = −1.759; *p* = 0.079). The effect size is strong (*r* = 0.44).

## Discussion

The current study tested a musical feedback system called Jymmin^®^ with older adults. The hypotheses that musical engagement during a fitness work-out would increase the duration of exercise and decrease perceived subjective effort about the exercise duration were examined. Based on previous findings, it was expected that an increase in mood would be found as an effect of musical feedback exercise.

The results demonstrate that during Jymmin^®^, participants could work out significantly longer than during the control condition where participants trained while passively listening to music (conventional work-out). Note that participants were monitored with one experimenter each to ensure they performed exercise movements physiologically correct and performed continuously without taking breaks to not corrupt the parameter duration of the work-out. It has previously been shown that Jymmin^®^ has an effect on perceived exertion and seemed to have increased muscular effectivity such that less oxygen was needed to perform the same physical exercises (Fritz et al., 2013a). The current findings show that perceived exertion did not differ between the conventional work-out condition and the Jymmin^®^ condition even though participants worked out significantly longer during the Jymmin^®^ condition. This is probably because they perceived the music feedback work-out as less exhausting and were therefore willing and able to continue longer with the work-out. Both subjective experience and physiological parameters play a crucial role in sustaining an intense physical effort. As shown previously, Jymmin^®^ increased muscular effectivity such that the muscles were more relaxed during the exercise and needed less oxygen for the same amount of work (Fritz et al., 2013a).

Furthermore, an increased pain tolerance, probably related to increased endorphin levels, was observed as an effect of a Jymmin^®^ intervention, compared to a control condition where a similar work-out was performed during only passively listening to music (Fritz et al., 2018). Such physiological effects may also have increased endurance in terms of work out duration. A further relevant psychological mechanism and explanation for the current results may be the attentional focus (Terry et al., 2020). Dissociation or purposeful focusing away from unpleasant feelings of pain or fatigue is a frequently used strategy in exercising. External stimuli such as music and virtual reality applications (IJsselsteijn et al., 2004) are designed to focus away from internal body sensations during exercise, causing less focus on distress cues and possibly leading to a lower rate of perceived exertion. Furthermore, real-time auditory feedback on movement provides additional information about the movement outcome. In music-supported therapy, each movement is associated with feedback, and thus information about the quality of timing of the movement is provided constantly. Because Jymmin^®^ translates movement directly into musical sound, participants get continuous and immediate auditory feedback related to timing.

However, an issue that has to be addressed in relation to an increased work-out duration during Jymmin^®^, is the number of repetitions. This was actually lower during music feedback work-out than during the conventional work-out. This corresponds to a greater degree of isometric contractions (muscle actively held at fixed length) during the music feedback work-out. While the strain of isometric contractions on the muscle does not generally differ concerning amplitude, such a less stereotypic contraction pattern during music feedback training may have supported them to proportion their energetic reserves during training better, a process called pacing. Pacing is relevant to exercise therapy and health sports, given that it can improve control competence and compliance, and has been suggested to be especially beneficial for cardiac prevention and rehabilitation to self-regulate training intensity, minimizing health risks (Thiel et al., 2018). Such a less stereotypic contraction pattern during the music feedback sports may thus have been physiologically beneficial.

Music, especially when selected according to its motivational qualities, renders mood and feeling state more positive across various exercise modalities and tasks (Karageorghis et al., 2010; Terry et al., 2012). This positive effect appears to hold regardless of exercise intensity or the synchronicity of the music. Well-selected music is associated with increases in positive affect, and it has been proposed that the positive effects of music on feeling states can lead to an increased adherence to exercise (Karageorghis et al., 1999). Following previous research presenting evidence for a positive effect of Jymmin^®^ on mood, in a second hypothesis, we predicted the same would occur here with older adults. We provided our participants with a mood questionnaire (MDMQ by Steyer et al., 2003) before and after training. This questionnaire assesses the current mental state with three bipolar designed subscales (good/bad mood, alertness/fatigue, calmness/restlessness). Comparing the differences between Jymmin^®^ and the control condition showed no effects and was inconsistent with our previous research with a younger population (Fritz et al., 2013a). This finding may be related to differences in the emotion processing of older adults; furthermore, the much larger sample size in the previous study with younger participants investigating mood changes may play a role. Mood changes evoked by music will also relate to certain qualities of the music, for example if the music is happy or sad etc. The musical styles preferred by older adults may have had different effects compared to the work-out music applied to the younger population in the previous experiment. As it has been noted, the type and quality of the music in an intervention will produce different effects and should therefore be chosen carefully (Marks and Landaira, 2015). For example, in the context of promoting healthy aging, specific musical features may exert a more substantial stress-lowering effect (Möckel et al., 1994). However, it has been suggested that if combined with physical training, musical style may be surprisingly irrelevant (Madison et al., 2013). It has previously been demonstrated that as an effect of the musical agency during the strenuous work-out, participants experienced a positivity bias about the aesthetic quality of the music. In other words, participants would assess music as more beautiful when producing it themselves during a work-out than when listening passively (Fritz et al., 2016). This effect has been labeled the band effect and seems highly beneficial at motivating groups to physical exercise where individuals would under a non-exercise context have a different musical taste (because they, e.g., have a different musical background).

To evaluate Jymmin^®^, participants were asked to rate the perceived music, which they were playing while working out as well as the passively presented music. The participants perceived the music as quite neutral and did not prefer one or the other. The meta-analysis by Terry et al. (2020) revealed that the type of music does not play a fundamental role as well as music and movement are synchronous or asynchronous. More relevant in music perception seems to be the tempo. The magical number of human activation is 120 bpm since human movement and music perception are somehow bound at that tempo (Terry et al., 2020). The theory suggests a greater information processing capacity for external stimuli at low-to-moderate intensities (Terry et al., 2020). At high intensities those cues might get diminished by the overwhelming influence of physiological load.

It has been shown that music can lead to measurable benefits in terms of lowered ratings of perceived exertion and an ergogenic effect (Karageorghis and Priest, 2012). Active music making in combination with physical exercises when performed in an adequate setup may produce even stronger effects. Making music involves sensory systems and the motor system and a wide variety of higher-order cognitive processes (Herholz and Zatorre, 2012). The Jymmin^®^ activity can be regarded as both, a physical and cognitive training. Therefore, it seems to be a very useful method in promoting healthy aging in older adults.

## Limitations

It should be noted that the results of this study are based on a relatively small sample size. However, it has been rather difficult to guarantee whether all subjects performed the exercises to exhaustion. It is for example possible that participants, even though they were properly instructed and had each an experimenter to monitor their continuously physiologically correct exercise execution, stopped doing the exercises due to other factors besides endurance, such as a lack of motivation.

It may be beneficial to systematically choose musical styles for older adults according to their musical taste. While it has previously been shown that actively engaging in playing music during physical exercise will increase the aesthetic appreciation of the music, asking only for musical taste as has been done in the current study, may not be the best parameter to assess. Musical taste may be something quite stable over time, while aesthetic musical appreciation may be more variable and thus a better parameter to ask for. In a second step, a long-term study needs to be addressed to shed light into the effect of such musical feedback training in older adults to verify our first promising results.

## Conclusion

In conclusion, the current data demonstrate an increase in work-out duration as an interaction between musical control and physical exercise. Participants could do physical exercise for a longer duration during a music feedback physical exercise compared to a conventional exercise passively listening to music, while showing a similar level of exhaustion in both conditions in an all out exercise intervention. Especially the combination of Body Spider and Jymmin^®^ is a great trainings tool for elderly adults in group settings (in sport clubs, retirement homes or nursing homes) and should be considered in studies. The Body Spider is foldaway and does not take too much space. This fitness machine offers six seats for sitting exercises and six positions for standing exercises. The resistance-robes of the Body Spider guarantee for a safe and guided execution of the exercises. This seems to be perfect for elderly adults and facilitate the organization of physical exercise in groups for trainers or supervisors. The active music making component covered by Jymmin^®^ leads to joyful training surrounding and sensory stimulation, social interaction and communication, which is highly important in older age. It is important that elderly adults, or users should first try to pull the robes in different ways (fast, slow, holding it for some seconds) to perceive the change in the musical structure and get familiar with this tool. We recommend, that two participants should execute a familiarization session (like we did here in this feasibility study) to understand that they are playing a musical piece, while working out. The constant beat of 120 bpm, like we have chosen, is recommendable! The combination of Body Spider and Jymmin^®^ is a promising training tool for elderly adults, which could contribute to maintain or improve the health status.

## Data availability statement

The raw data supporting the conclusions of this article will be made available by the authors, without undue reservation.

## Ethics statement

The studies involving human participants were reviewed and approved by Ethics Committee of the Medical Faculty Otto von Guericke University (No. 193/18). The patients/participants provided their written informed consent to participate in this study.

## Author contributions

KW, TF, AP, and LS conceived and planned the study and experiments. AP, TF, and LS conducted the intervention. TF, KR, AP, and LS analyzed the data. TF, KR, AP, KW, LS, and AV contributed to the interpretation of the results. KR and TF took the lead in writing the manuscript. All authors contributed to the article and approved the submitted version.

## Conflict of interest

One of the contributing authors TF has been involved at founding the startup Jymmin GmbH that has the aim to develop further the music feedback technology. TF is also a shareholder in the Jymmin GmbH. The remaining author declares that the research was conducted in the absence of any commercial or financial relationships that could be construed as a potential conflict of interest.

## Publisher's note

All claims expressed in this article are solely those of the authors and do not necessarily represent those of their affiliated organizations, or those of the publisher, the editors and the reviewers. Any product that may be evaluated in this article, or claim that may be made by its manufacturer, is not guaranteed or endorsed by the publisher.
