# Corrigendum: Musical feedback system Jymmin^®^ leads to enhanced physical endurance in the elderly—A feasibility study

**DOI:** 10.3389/fspor.2022.1019240

**Published:** 2022-09-08

**Authors:** Kathrin Rehfeld, Thomas Hans Fritz, Alexander Prinz, Lydia Schneider, Arno Villringer, Kerstin Witte

**Affiliations:** ^1^Institute for Sport Science, Otto-von-Guericke University Magdeburg, Magdeburg, Germany; ^2^Department of Neurology, Max Planck Institute for Human Cognitive and Brain Sciences, Leipzig, Germany; ^3^Institute for Psychoacoustics and Electronic Music (IPEM), Ghent, Belgium

**Keywords:** Jymmin^®^, seniors, physical exercise, aging, musical feedback system

In the published article, there was an error regarding the affiliations for “Thomas Hans Fritz.” As well as having affiliation 2, he should also have “Institute for Psychoacoustics and Electronic Music (IPEM), Ghent, Belgium.”

In the published article the conflict of interest was not appropriately stated. The conflict of interest statement has now been inserted and should read:

## Conflict of interest

One of the contributing authors TF has been involved at founding the startup Jymmin GmbH that has the aim to develop further the music feedback technology. TF is also a shareholder in the Jymmin GmbH. The authors apologize for this error and state that this does not change the scientific conclusions of the article in any way. The original article has been updated.

## Publisher's note

All claims expressed in this article are solely those of the authors and do not necessarily represent those of their affiliated organizations, or those of the publisher, the editors and the reviewers. Any product that may be evaluated in this article, or claim that may be made by its manufacturer, is not guaranteed or endorsed by the publisher.

